# Effect of penehyclidine hydrochloride on inflammatory response and oxidative stress in rats with cardiopulmonary bypass related-lung injury

**DOI:** 10.1590/acb370406

**Published:** 2022-06-27

**Authors:** Man He, Yilin Zhao, Shiyong Li, Ailin Luo, Hong Chen

**Affiliations:** 1 MD. Huazhong University of Science and Technology – Tongji Medical College – Tongji Hospital – Department of Anesthesiology – Wuhan, China.

**Keywords:** Cardiopulmonary Bypass, Lung Injury, Inflammation, Oxidative Stress

## Abstract

**Purpose:**

To investigate the protective effect of penehyclidine hydrochloride (PHC) on cardiopulmonary bypass (CPB)-related lung injury in rats.

**Methods:**

Thirty-six rats were divided into control, CPB and PHC groups. The CPB model was established in CPB and PHC groups. In PHC group, 2-mg/kg PHC was added to the pre-filling solution for CPB modeling. At 30 min before CPB (T1), immediately after left hilar opening (T2) and end of experiment (T3), the hemodynamic indexes, blood gas indexes, serum inflammatory factors, lung wet-day ratio and water content and lung tissue oxidative stress indexes were determined.

**Results:**

At T2 and T3, compared with CPB group, in PHC group the heart rate and mean arterial pressure increased significantly, the oxygenation index increased significantly, the respiratory index decreased significantly, and the lung wet-day ratio and water content decreased significantly. At T3, compared with CPB group, in PHC groups the serum tumor necrosis factor α, interleukin 6 and interleukin 1β levels decreased significantly, the lung tissue superoxide dismutase level increased significantly, and the myeloperoxidase and malondialdehyde levels decreased significantly.

**Conclusions:**

PHC treatment can alleviate the CPB-related lung injury in rats. The mechanisms may be related to its reducing inflammatory response and resisting oxidative stress.

## Introduction

Cardiopulmonary bypass (CPB) is a life support technology that uses a series of special artificial devices to temporarily replace the human heart and lung for blood circulation and gas exchange^1^. During CPB, due to the change of pulsatile perfusion to advection perfusion, the physical, chemical, and biological properties of blood vary greatly, which is easy to cause the microcirculation disorder^2^. The lungs are one of the most sensitive organs to CPB. CPB-related lung injury is one of the most common complications of cardiac surgery. Its mechanism may be related to the systemic inflammatory reaction caused by the contact between blood and artificial pipes and instruments and the lung ischemia-reperfusion injury during CPB^3^^,^ 4. Patients with CPB-related lung injury are manifested by respiratory mechanic changes, decreased lung compliance and hypoxemia, and these complications may be fatal for the patients. At the same time, the resulting pulmonary dysfunction will prolong the hospital stay, increase the treatment cost, and reduce the survival rate of patients^5^.

Penehyclidine hydrochloride (PHC) belongs to the anticholinergic drugs, which can selectively block the M1, M3, N1 and N2 receptors, reduce the permeability of capillary wall, reduce the exudation of inflammatory factors, stabilize the membrane structure of subcellular organelles such as lysosomes, inhibit the release of shock factors, thus exerting the cytoprotective effect^6^^,^ 7. PHC is the most widely used anesthetic adjuvant in clinic. Recent studies have shown that PHC not only has the effect of anti-cholinergic receptor, but also can regulate the microcirculation, reduce the oxidative stress, and decrease the apoptosis^8^.

This study was designed to investigate the protective effect of PHC on CPB-related lung injury in rats and the related mechanisms.

## Methods

This study was carried out in strict accordance with the recommendations in the Guide for the Care and Use of Laboratory Animals of the National Institutes of Health. The animal use protocol has been reviewed and approved by the Institutional Animal Care and Use Committee (IACUC) of Huazhong University of Science and Technology.

### Animal grouping and treatment

Thirty-six healthy adult Sprague Dawley rats (350-450 g; Shanghai Slake Experimental Animal Co., Ltd., Shanghai, China) were adaptively fed under condition of 12 h/12 h light/dark cycle, temperature of 22 ± 2°C and humidity of 50 ± 5% for one week. Then, the rats were randomly divided into control group, CPB group and PHC group, with 12 rats in each group.

### Establishment of cardiopulmonary bypass

The rats in the three groups were anesthetized by intraperitoneal injection of 1% pentobarbital sodium (Sigma-Aldrich Co., Shanghai, China), with dose of 50 mg/kg. The catheterization of the caudal vein, the left femoral artery, the bilateral femoral veins and the right common carotid artery was performed, respectively. The endotracheal intubation was performed with a special tube, which was connected with the small animal ventilator (Shanghai Yuyan Scientific Instrument Co., Ltd., Shanghai, China). The left femoral artery was connected to the biological signal acquisition and processing system (Anhui Zhenghua Biological Instrument Equipment Co., Ltd., Huaibei, China) to monitor the vital signs. After the pre-filling of CPB pipeline, 3-mg/kg heparin (Sigma-Aldrich Co., Shanghai, China) was injected into the caudal vein. Then, using bilateral femoral veins as the drainage end, the right common carotid artery was connected with CPB pipeline as the reflux end. After the activated clotting time of whole blood was greater than 480 s, the CPB machine (Xi’an Xijing Medical Supplies Co., Ltd., Xi’an, China) started to work. The perfusion flow rate was maintained as 40 mL/(kg·min). After working for 10 min, the chest was opened to expose the left hilar. The left hilar was clamped for one-lung ventilation. After continued work for 45 min, the left hilar was opened. After 30 min, the protamine (Sigma-Aldrich Co., Shanghai, China) was injected to neutralize the excess heparin. After the vital signs of rats were stable, the CPB was stopped. After 90 min, the experiment was completed. In PHC group, 2-mg/kg PHC was added to the pre-filling solution, and in CPB group the same amount of normal saline was added to the pre-filling solution. The control group only underwent the endotracheal intubation and thoracotomy, without other treatment.

### Hemodynamic measurement

At 30 min before CPB (T1), immediately after left hilar opening (T2) and the end of experiment (T3), the hemodynamic indexes including heart rate (HR) and mean arterial pressure (MAP) were recorded using the biological signal acquisition and processing system.

### Blood gas analysis

At T1, T2 and T3, the femoral artery blood of each group was taken. The blood gas analysis was performed using blood gas analyzer (Johnson & Johnson Medical Equipment Co., Ltd., Suzhou, China). The oxygenation index (OI) and respiratory index (RI) were calculated.

### Determination of serum inflammatory factors

At T3, the femoral artery blood of each group was taken. The blood was centrifuged at 4°C and 3,000 rpm for 15 min. The serum was obtained. The serum tumor necrosis factor α (TNF-α), interleukin 6 (IL-6) and interleukin 1β (IL-1β) levels were determined using the enzyme-linked immunosorbent assay. The determination procedures were according to the instructions of the kits (Sigma-Aldrich Co., Shanghai, China).

### Determination of lung wet-day ratio and water content

At T3, the rats were sacrificed. The left lung was isolated. The upper lobe tissue of lung was taken and placed on the balance to record the wet weight. Then, the lung tissue was dried at 80°C for 72 h, and the dry weight was recorded. The lung wet-day ratio and the lung water content (wet weight - day weight) / wet weight were calculated.

### Determination of lung tissue oxidative stress indexes

The lower lobe tissue of left lung was taken and weighed, and then were cut into pieces on the ice bath. After adding the pre-cooled normal saline, the tissue homogenate was prepared. After centrifuging at 4°C and 3,000 rpm for 15 min, the supernatant was obtained. The myeloperoxidase activity was determined by colorimetry. The superoxide dismutase (SOD) activity was determined by xanthine oxidase method. The malondialdehyde content was determined by thiobarbituric acid method. The determination procedures were according to the instructions of the kits (Sigma-Aldrich Co., Shanghai, China).

### Statistical analysis

Statistical Package for the Social Sciences (SPSS) software was used for statistical analysis. The data were presented as mean ± standard deviation. The comparison among three time points and among three groups was made by one-way analysis of variance (ANOVA) followed by least significant difference (LSD)-t test. P < 0.05 presented the statistically significant difference.

## Results

### Hemodynamic indexes

**Figure 1:**
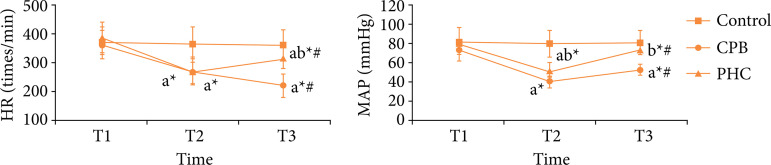
Hemodynamic indexes in three groups.

There was no significant difference in HR or MAP among three time points in control group (P > 0.05). Compared with T1, at T2 and T3 the HR and MAP in CPB and PHC groups decreased significantly, respectively (P < 0.05). Compared with T2, at T3 in CPB group the HR decreased significantly (P < 0.05), and the MAP increased significantly (P < 0.05); in PHC group the HR and MAP increased significantly, respectively (P < 0.05). At T1, there was no significant difference in HR or MAP among three groups (P > 0.05). At T2, compared with control group, in CPB and PHC groups the HR and MAP decreased significantly, respectively (P < 0.05); compared with CPB group, in PHC group the MAP increased significantly (P < 0.05). At T3, compared with control group, in CPB and PHC groups the HR and MAP decreased significantly, respectively (P < 0.05); compared with CPB group, in PHC group the HR and MAP increased significantly, respectively (P < 0.05) (Fig. 1).

*P < 0.05 *vs.* T1; ^#^P < 0.05 *vs*. T2; ^a^P < 0.05 *vs*. control group; ^b^P < 0.05 *vs.* CPB group; HR: heart rate; MAP: mean arterial pressure; CPB: cardiopulmonary bypass; PHC: penehyclidine hydrochloride.

### Blood gas indexes

**Figure 2:**
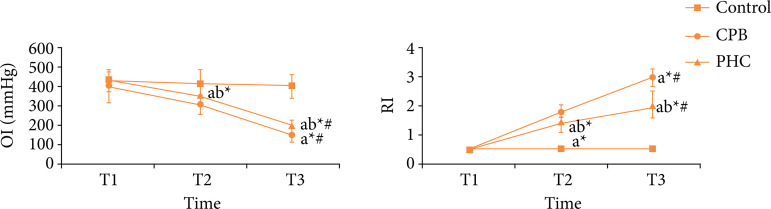
Blood gas indexes in three groups.

The OI and RI had no significant difference among three time points in control group, respectively (P > 0.05). Compared with T1, at T2 and T3 in CPB and PHC groups the OI decreased significantly, respectively (P < 0.05), and the RI increased significantly, respectively (P < 0.05). Compared with T2, at T3 in CPB and PHC groups the OI decreased significantly, respectively (P < 0.05), and the RI increased significantly (P < 0.05). At T1, there was no significant difference in OI or RI among three groups (P > 0.05). At T2 and T3, compared with control group, in CPB and PHC groups the OI decreased significantly, respectively (P < 0.05), and the RI increased significantly, respectively (P < 0.05); compared with CPB group, in PHC group the OI increased significantly (P < 0.05), and the RI decreased significantly (P < 0.05) (Fig. 2).

*P < 0.05 *vs.* T1; ^#^P < 0.05 *vs*. T2; ^a^P < 0.05 *vs*. control group; ^b^P < 0.05 *vs.* CPB group; OI: oxygenation index; RI: respiratory index; CPB: cardiopulmonary bypass; PHC: penehyclidine hydrochloride.

### Lung wet-day ratio and water content

**Figure 3:**
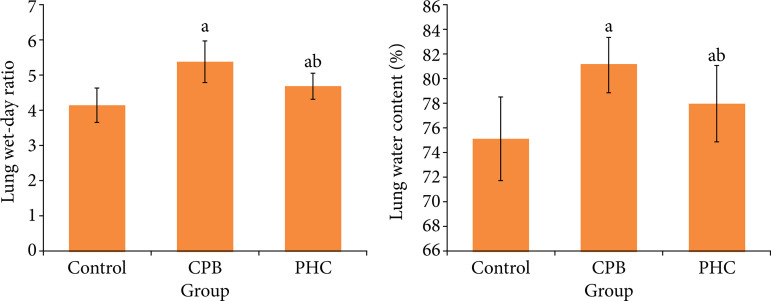
Lung wet-day ratio and water content in three groups.

^a^P < 0.05 *vs*. control group; ^b^P < 0.05 *vs.* CPB group; CPB: cardiopulmonary bypass; PHC: penehyclidine hydrochloride.

At T3, compared with control group, in CPB and PHC groups the lung wet-day ratio and water content increased significantly, respectively (P < 0.05). Compared with CPB group, in PHC group the lung wet-day ratio and water content decreased significantly, respectively (P < 0.05) (Fig. 3).

### Serum inflammatory factors

**Figure 4:**
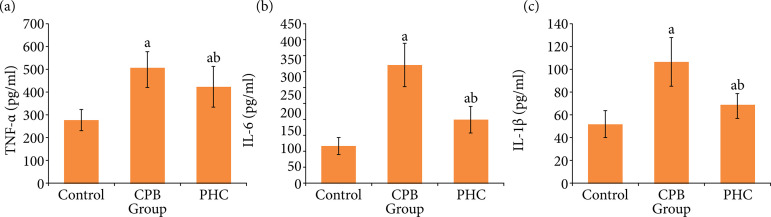
Serum inflammatory factors in three groups.

^a^P < 0.05 *vs*. control group; ^b^P < 0.05 *vs.* CPB group; CPB: cardiopulmonary bypass; PHC: penehyclidine hydrochloride; TNF-α: tumor necrosis factor α; IL-6: interleukin 6; IL-1β: interleukin -1β.

At T3, compared with control group, in CPB and PHC groups the serum TNF-α, IL-6 and IL-1β levels increased significantly, respectively (P < 0.05). Compared with CPB group, in PHC group each index decreased significantly (P < 0.05) (Fig. 4).

### Lung tissue oxidative stress indexes

**Figure 5:**
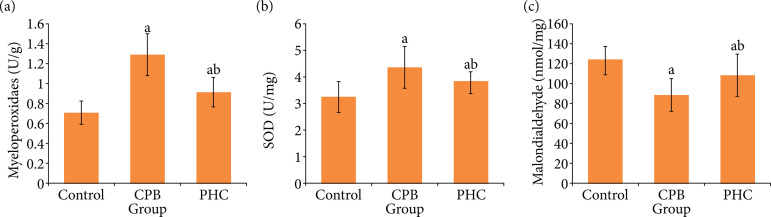
Lung tissue oxidative stress indexes in three groups.

^a^P < 0.05 *vs*. control group; CPB: cardiopulmonary bypass; PHC: penehyclidine hydrochloride; SOD: superoxide dismutase.

At T3, compared with control group, in CPB and PHC groups the lung tissue SOD level decreased significantly, respectively (P < 0.05), and the myeloperoxidase and malondialdehyde levels increased significantly, respectively (P < 0.05). Compared with CPB group, in PHC group the SOD level increased significantly (P < 0.05), and the myeloperoxidase and malondialdehyde levels decreased significantly, respectively (P < 0.05) (Fig. 5).

## Discussion

CPB is a necessary condition and safeguard measure for open heart surgery. In CPB, the artificially change of cardiopulmonary perfusion mode can lead to the drastic changes in blood composition and hemodynamics, which induces the systemic inflammatory stress response^9^. The lungs are one of the most sensitive organs to systemic inflammatory stress response^10^. The research on CPB-related lung injury and the protection have always been a hot spot^11^. Therefore, it is very important to prevent the CPB-related lung injury.

In this study, the rat CPB model with beating heart was established, which excludes the effect of cardiac arrest on lung tissue and largely simulated the lung injury of CPB in non-physiological circulation state^12^. HR and MAP can reflect the hemodynamic state of body. Among multiple respiratory indexes, OI and RI are the most direct and accurate indexes to reflect the degree of lung injury. The lung wet-day ratio and water content reflect the edema of lung tissues. Results of this study found that, at T2 and T3, compared with CPB group, in PHC group the HR and MAP increased significantly, the OI increased significantly, the RI decreased significantly, and the lung wet-day ratio and water content decreased significantly. This suggests that the PHC pretreatment can stabilize the hemodynamics, reduce the lung edema, and improve the lung function in rats receiving CPB.

Systemic inflammatory response is one of the mechanisms of CPB-related lung injury^13^. TNF-α is the most sensitive indicator of inflammatory response, and the initiating factor of various pro-inflammatory factors^14^. IL-6 is one of interleukin family members activated by TNF-α and the key relay factor for amplifying and accelerating the inflammatory response^15^. IL-1β is an early inflammatory cytokine that activates the nuclear factor-aB to produce cytokines, thus participating in the initiation of inflammatory response^16^. In our study, compared with control group, in CPB and PHC groups the serum TNF-α, IL-6 and IL-1β levels increased significantly. Compared with CPB group, in PHC group each index decreased significantly. This indicates that the PHC treatment may reduce the systemic inflammatory response, thus alleviating the CPB-related lung injury in rats.

Oxidative stress is closely related to the CPB-related lung injury^17^^,^ 18. SOD is a very important antioxidant enzyme system in the process of oxidative stress. It can help the body to clear the superoxide anion free radicals^19^. Malondialdehyde is an important degradation product of lipid peroxidation. The content of MDA can indirectly reflect the damage degree of tissues and cells attacked by free radicals^20^. Myeloperoxidase is an important member of heme peroxidase superfamily. It is the activation marker of neutrophils in the body^21^. Myeloperoxidase can also cause the oxidative stress and tissue damage^22^. Therefore, these three indexes are the very valuable indicators to reflect the degree of oxidative stress damage. In our study, compared with CPB group, in PHC group the lung tissue SOD level increased significantly, and the myeloperoxidase and malondialdehyde levels decreased significantly. It is suggested that PHC treatment may reduce the degree of oxidative stress, which is related to its alleviation of CPB-related lung injury in rats.

## Conclusions

The PHC treatment can alleviate the CPB-related lung injury in rats. The mechanisms may be related to its reducing inflammatory response and resisting oxidative stress. The other action mechanisms still need to be further clarified.
